# The Influence of Photoreceptor Size and Distribution on Optical Sensitivity in the Eyes of Lanternfishes (Myctophidae)

**DOI:** 10.1371/journal.pone.0099957

**Published:** 2014-06-13

**Authors:** Fanny de Busserolles, John L. Fitzpatrick, N. Justin Marshall, Shaun P. Collin

**Affiliations:** 1 The School of Animal Biology and The UWA Oceans Institute, The University of Western Australia, Crawley, Australia; 2 Red Sea Research Centre, King Abdullah University of Science and Technology, Thuwal, Saudi Arabia; 3 Centre for Evolutionary Biology, School of Animal Biology, The University of Western Australia, Crawley, Australia; 4 Computational and Evolutionary Biology, Faculty of Life Sciences, University of Manchester, Manchester, United Kingdom; 5 Sensory Neurobiology Group, Queensland Brain Institute, University of Queensland, Brisbane, Australia; Lund University, Sweden

## Abstract

The mesopelagic zone of the deep-sea (200-1000 m) is characterised by exponentially diminishing levels of downwelling sunlight and by the predominance of bioluminescence emissions. The ability of mesopelagic organisms to detect and behaviourally react to downwelling sunlight and/or bioluminescence will depend on the visual task and ultimately on the eyes and their capacity for detecting low levels of illumination and intermittent point sources of bioluminescent light. In this study, we investigate the diversity of the visual system of the lanternfish (Myctophidae). We focus specifically on the photoreceptor cells by examining their size, arrangement, topographic distribution and contribution to optical sensitivity in 53 different species from 18 genera. We also examine the influence(s) of both phylogeny and ecology on these photoreceptor variables using phylogenetic comparative analyses in order to understand the constraints placed on the visual systems of this large group of mesopelagic fishes at the first stage of retinal processing. We report great diversity in the visual system of the Myctophidae at the level of the photoreceptors. Photoreceptor distribution reveals clear interspecific differences in visual specialisations (areas of high rod photoreceptor density), indicating potential interspecific differences in interactions with prey, predators and/or mates. A great diversity in photoreceptor design (length and diameter) and density is also present. Overall, the myctophid eye is very sensitive compared to other teleosts and each species seems to be specialised for the detection of a specific signal (downwelling light or bioluminescence), potentially reflecting different visual demands for survival. Phylogenetic comparative analyses highlight several relationships between photoreceptor characteristics and the ecological variables tested (depth distribution and luminous tissue patterns). Depth distribution at night was a significant factor in most of the models tested, indicating that vision at night is of great importance for lanternfishes and may drive the evolution of their photoreceptor design.

## Introduction

As sunlight penetrates the water column of the ocean, it is absorbed and scattered leading to a rapid decrease in intensity with depth, and in the mesopelagic zone (200 to 1000 m) very low levels of residual downwelling sunlight remain. Residual light in the mesopelagic zone is of constant colour and direction but of exponentially diminishing intensity, creating an extended visual scene (vertically and horizontally), where silhouettes of organisms can be distinguished, against a light background, when viewed from below. The intensity gradient of downwelling sunlight might be also used to maintain a specific depth during the day [1], be used for orientation for vertical migration [2], to camouflage the outline of the body by counter-illumination [3], [4], and/or to detect the presence of other animals above, since they would cast a detectable shadow. However, most mesopelagic organisms also produce and/or emit bioluminescent signals, which can be of different spectral composition, intensity and duration [5] and are used in the mediation of a number of important behaviours including the detection of prey and predator, the attraction and/or avoidance of other organisms and the communication with conspecifics [6], [7]. Therefore, the ability of mesopelagic organisms to detect and behaviourally react to bioluminescent emissions and/or downwelling sunlight will depend on the visual task and ultimately on the eyes and their capacity for detecting low levels of illumination and intermittent point sources of bioluminescent light.

Due to the low light environment and the predominance of small bioluminescent flashes, the eyes of mesopelagic fishes are more sensitive than their shallow water counterparts, relying less on acuity to resolve fine visual detail. Specialisations to increase the sensitivity of the eye include extending the visual field to allow the capture of as many photons as possible. This can be achieved with the help of tubular-shaped eyes, which are directed upwards to maximise the capture of the downwelling sunlight [8]–[10], with an increased size of both the eye and the pupillary aperture [11] and/or the presence of an aphakic gap, a region of the pupillary aperture that allows light from a specific part of the visual field to reach the retina without necessarily passing through the lens [8], [12]. Visual adaptations for increasing sensitivity at the level of the retina include 1. the tapetum lucidum, a mirror-like structure which sits at the back of the eye and increases light absorption [13], [14], 2. high numbers of long rod photoreceptors, which are specialised for scotopic vision [9] often arranged in multibanks [8], [9] which, in addition to enhancing the chance of photon capture, may also allow colour vision in single pigment species or at least provide hue discrimination to potentially break camouflage by counterillumination [15] and 3. most mesopelagic fishes possess a single visual pigment within their photoreceptors that closely matches both the predominant wavelengths of the downwelling sunlight and the spectral emission of most bioluminescence [16]–[18].

The lanternfish family (Myctophidae) is one of the most abundant groups of mesopelagic fishes in the world [19] with more than 250 representative species from 33 genera [20]. They are present worldwide and live at the surface down to depths exceeding 1000 m, thereby playing a major role in oceanic ecosystems by transferring energy to deeper levels through their daily vertical migrations. These vertical migrations are for the purposes of feeding but there is large inter- and intra-specific variability [21] depending on life stage and season [22]. Like most mesopelagic organisms, lanternfishes are bioluminescent and produce their own light through a luciferin-luciferase reaction [23], [24], which takes place within two kinds of bioluminescent structures; the photophores found on the ventral and ventrolateral parts of the body and the luminous organs and tissue patches present on the head, body and/or tail. While the photophores are thought to play a major role in camouflage by counter-illuminating the underside of the body [25], luminous organs are thought to play several different roles in intra- and interspecific communication, distraction or illumination [26]. Luminous tissue patterns are highly variable between species and, in some cases, are sexually dimorphic, indicating that the visual system must play an important role in finding reproductive partners. Overall, the abundance of lanternfishes and the high level of variability in their depth distribution, vertical migration patterns and luminous tissue dimorphisms, make this group an important model for visual adaptation studies. However, very little information is available about their visual capabilities with respect to their photic environment.

Like other mesopelagic organisms, lanternfishes possess well-developed eyes [27], which appear to be adapted to enhance sensitivity due to specialisations such as an aphakic gap [28], [29], a tapetum lucidum [29], [30], a pure rod retina [29]–[32], a high density of photoreceptors [30]–[32], and visual pigments tuned to their ambient light environment of downwelling sunlight and bioluminescent flashes [18], [33], [34], [35], [36]. Recently, de Busserolles et al. [29] highlighted the great interspecific variability in eye size within the Myctophidae by investigating the relationship between eye size (corrected for body size) and ecological variables (i.e. depth distribution and luminous tissue pattern). They hypothesized that species living at greater depths and/or relying less on bioluminescent emissions for vision will consequently have smaller eyes. However, they did not find any relationship between eye size and any of the ecological variables tested but instead indentified the presence of a strong phylogenetic signal [27]. It is therefore apparent that the visual capabilities of a species may not be solely assessed by the size of the eye and that a number of other physical factors (i.e. biophysical properties of the photoreceptors) in addition to visual stimuli need to be considered in order to assess the relationship between vision and ecology. In this study, we investigate the diversity of the myctophid visual system, concentrating specifically on the photoreceptor cells by examining their size, arrangement, topographic distribution and contribution to optical sensitivity in 53 different species from 18 genera. We also examine the influence(s) of both phylogeny and ecology on these photoreceptor variables in order to understand the constraints placed on the visual systems of this large group of mesopelagic fishes at the first stage of retinal processing.

## Materials and Methods

### Ethics statement

Samples were obtained from several research cruises in the Coral Sea (RV *Cape Ferguson*) under the following collection permits: Coral Sea waters (CSCZ-SR-20091001-01), Commonwealth waters (AU-COM2009051), GBRMPA (G09/32237.1) and Queensland Fisheries (133805), (Marshall, AEC # SNG/080/09/ARC), and in the Peru-Chile trench (FS *Sonne*, sampling permits obtained by the Chief Scientist, University of Tübingen). For all specimens, sampling was carried out following the guidelines of the NH&MRC Australian Code of Practice, under a University of Western Australia Animal Ethics protocol (RA/3/100/917). Additional specimens from Western Australian waters, the Western Mediterranean Sea and the Bay of Biscay were acquired through collaborators [27] and did not require UWA collection or animal ethics permits.

### Samples

The eyes of 53 different species of lanternfishes from 18 genera were analysed in this study. While most of the samples are registered as voucher specimens at the Australian Museum in Sydney, Australia, further taxonomic analyses need to be carried out for five of our study species to positively confirm identification (*Lampanyctus vadulus*, *Myctophum spinosum*, *Nannobrachium cf. nigrum*, *Symbolophorus cf. boops*, *Triphoturus oculeus*, [27]).

Observations were performed on board and on fresh specimens when possible. For each individual, the standard length and rostro-caudal eye diameter were measured with digital callipers to an accuracy of 0.1 mm prior to dissection. The position of the aphakic gap (dorsal, nasal, ventral, temporal), when present, was noted and photographed onboard using a Canon digital camera mounted on an Olympus stereomicroscope (Model SZX10). Eyes were then enucleated, the cornea and lens dissected free of the posterior chamber and the lens diameter measured using digital callipers (to 0.1 mm). The appearance and colour of the tapetum lucidum was noted and the fundus of the eye was also photographed. The eyes, lens and cornea were all fixed in 4% paraformaldehyde in 0.1 M phosphate buffer (PFA, pH 7.4) or Karnovsky's fixative (2% paraformaldehyde, 2.5 glutaraldehyde in 0.1 M cacodylate buffer, pH 7.4) for at least 1 h and then stored in 0.1 M phosphate buffer. When specimens and their eyes were very small, the entire individual was fixed (as above) and the eyes dissected (post fixation) back in the mainland laboratory. When samples were acquired from collaborators, observations, measurements and dissections were all performed on postfixed tissue, with the eyes and bodies preserved in 5% buffered formalin or Karnovsky's fixative.

### Preparation of retinal wholemounts

Several eyes fixed in 4% PFA or Karnovsky's fixative were dissected in order to analyse the topographic distribution of photoreceptors across the retina. Wholemounts of the retina were prepared according to standard protocols [37]–[39]. Radial cuts were performed in order to flatten the eye and subsequently the retina *in toto* onto a glass slide, where the orientation was confirmed by making a small additional cut in the nasal or dorso-nasal part of the eye. The sclera, retinal pigment epithelium and tapetum (when present), were gently removed with the help of No. 3 watchmaker's forceps and a kolinsky hair paint brush. Each retinal wholemount was then processed following the protocol of Curcio et al. [40] to allow better visualisation of the photoreceptors. Briefly, the retina was quickly rinsed in distilled water and flat-mounted on a microscope slide, photoreceptor side facing up. The retinal wholemount was initially mounted in 100% dimethyl sulfoxide (DMSO) for 24 h to clear the tissue and re-mounted in 100% glycerol for topographic analysis.

### Stereological analysis and the construction of topographic maps

The topographic distribution of the photoreceptors was assessed using the optical fractionator technique [41] modified by Coimbra et al. [42], [43]. Briefly, the retinal wholemount was considered as a single section (section sampling fraction, ssf = 1) and since the photoreceptors are organised in a single layer in lanternfishes, the thickness sampling fraction (tsf) was fixed at 1. The outline of the retinal wholemount was then digitised using a x4 objective (numerical aperture 0.13) mounted on a compound microscope (Olympus BX50) equipped with a motorised stage (MAC5000, Ludl Electronics Products, USA), a digital video camera (MicroFIRE, OPTRONICS), and a computer running Stereo Investigator software (Microbrightfield, USA). Using a x100 oil immersion objective (numerical aperture 1.40), rod photoreceptors were randomly and systematically counted using the parameters listed in Table 1.

**Table 1:** Summary of the stereological parameters used for the topographic analyses of the photoreceptor cells in five lanternfish species.

Species	Individual	SL (mm)	Eye ø (mm)	Counting frame (µm×µm)	Grid (µm×µm)	Site numbers
*Bolinichthys longipes*	A	35.0	3.2	10 ×10	320×320	200
	B	36.8	3.4	10×10	340×340	204
	C	45.4	4.4	10×10	370×370	199
*Lampanyctus parvicauda*	A	54.2	3.3	10×10	270×270	198
	B	60.3	3.7	10×10	300×300	211
	C	65.2	4.5	10×10	350×350	209
*Myctophum brachygnathum*	A	65.7	7.1	5×5	850×850	107
	B	67.7	7.6	5×5	850×850	100
	C	68.3	7.7	5×5	850×850	97
*Diaphus brachycephalus*	A	?	?	15×15	270×270	205
*Nannobrachium idostigma*	B	72.2	3.9	15×15	280×280	197

**Figure 1:**
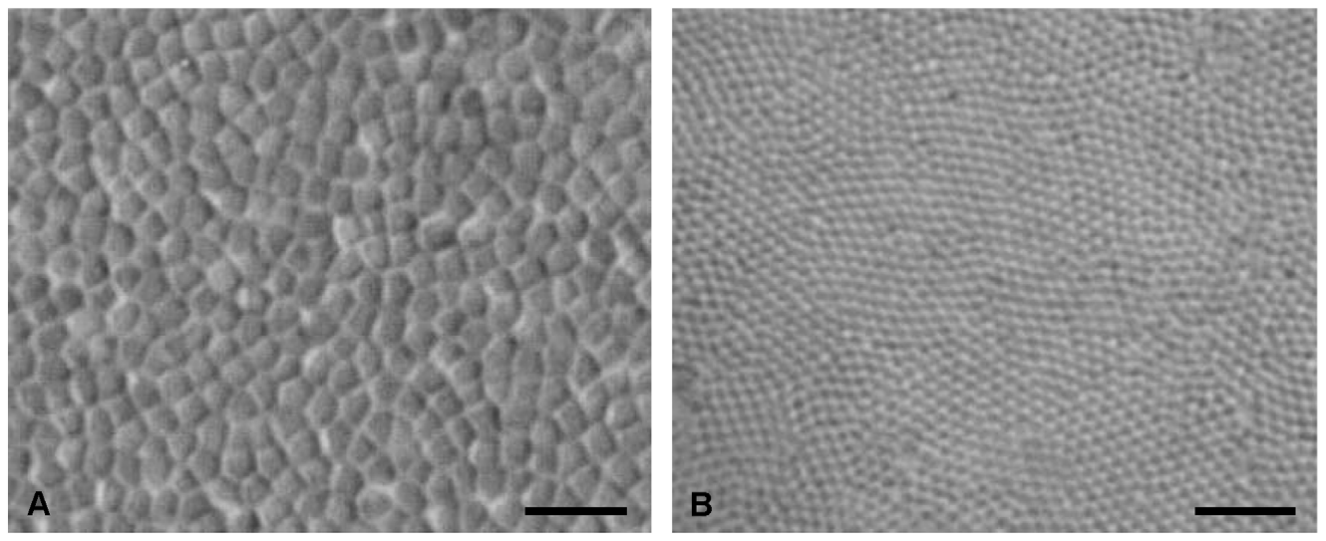
Wholemount view of the rod photoreceptors in *Diaphus brachycephalus* (A) and *Myctophum brachygnathum* (B). Scale bar = 10 µm.

Photoreceptor counts were very challenging and time consuming in lanternfishes due mainly to the small size of their rod photoreceptors (0.9 – 2.7 µm, Table S1), which were often beyond the optical limits of the light microscope. As a result, only five species were analysed in this study. The number of individuals per species analysed ranged from one to three due to the availability of samples and the quality of fixation, which was often outside of our control depending on the delay in preserving specimens once they reached the deck and/or the depth of capture. In order to maintain the same high level of sampling and the comparison of small and large individuals, the grid size was modified to allow the sampling of around 200 sites per retina and to achieve an acceptable Schaeffer coefficient of error (CE). The CE is a measure of the accuracy of the total number of cell estimates and is considered acceptable below 0.1 [44], [45]. For one species, *Myctophum brachygnathum*, photoreceptor counts was particularly challenging, even at the highest magnification offered by the stereology setup, due to their extremely small size (∼ 1 µm, Figure 1B). Consequently, a larger grid was used for this species, sampling only 100 sites per retina, but still achieving a CE <0.1, although sub-sampling in the area of high cell density was carried out for two individuals in order to compensate for the use of a larger counting frame.

Topographic maps of photoreceptor density were constructed using the statistical program R v.2.15.0 (R Foundation for Statistical Computing 2012) with the results exported from the Stereo Investigator Software according to Garza Gisholt et al. [46]. Garza Gisholt et al. [46] proposed several smoothing models to construct the iso-density maps and, for this study, we chose to use the Gaussian Kernel Smoother from the Spatstat package [47]. For each map, the sigma value was adjusted to the distance between points (i.e. grid size).

### Morphometric measurement of the photoreceptors

Outer and inner segment length and diameter were measured for a subset of the rod photoreceptors from transverse sections of the retina using light and electron microscopy. Samples fixed in Karnovsky's fixative were preferentially used, although when no samples preserved in Karnovsky's fixative were available, samples fixed in 4% PFA or in 5% buffered formalin were used. One eye per species was analysed. Depending on the size of the samples, the whole, half or a quadrant of the eye was processed. The tissue was postfixed for an hour with 1% osmium tetroxide in 0.15 M phosphate buffer, dehydrated through an alcohol and propylene oxide series and infiltrated with procure/araldite (ProSciTech). For light microscopy, semi-thin sections (1 µm) were cut with a glass knife using a LKB Bromma Ultratome NOVA. Sections were stained with an aqueous mixture of 0.5% Toluidine Blue and 0.5% borax, viewed with an Olympus BX50 compound light microscope and photographed using an Olympus DP70 digital camera. For transmission electron microscopy, thin sections (110 nm) were cut using a diamond knife, mounted on a 200 mesh copper grid and stained with Reynold's lead citrate. Examination of the sections was done using a JEOL 2100 transmission electron microscope operating at 120 kV and images taken using an 11 megapixel Gatan Orius digital camera.

All measurements were made from digital images using ImageJ 1.45 (National Institutes of Health, USA). To allow comparison between species, all measurements were taken in the central part of the retina, except for *Benthosema suborbitale*. In the case of *B. suborbitale*, which possesses a great variability in retinal thickness across the retina, with the ventral part being nearly double the thickness of the remaining retina [29], measurements were made in both the ventral and dorsal retinal regions. For all species, eight measurements were performed for each of the photoreceptor parameters and the average measure was reported. Please note that the term inner segment in this study refers only to the ellipsoid region of the rod photoreceptor.

In addition to the labour-intensive assessment of the density of rod photoreceptors using the stereological optical fractionator method in the retinal wholemounts of five different species, photoreceptor density was also estimated from a transverse section for all other species for interspecific comparison. Cells were counted, in a defined region of central retina, per unit of length and then converted in cells per m^2^. To confirm, that the densities calculated from retinal wholemounted material align with those using sections, we performed a direct comparison in the five species for which both data were available and reveal that there was close agreement.

### Optical sensitivity estimations

Optical sensitivity to downwelling light and to bioluminescent flashes was estimated separately for each species using direct measurements and data from the literature.

Sensitivity to downwelling light (extended sources, in units of µm^2^ sr), calculated at the level of single photoreceptors, was estimated using the formula from Land [48]:
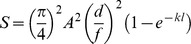



In the equation, *A* is the diameter of the pupil, *f* the focal length of the eye (lens radius x a Matthiessen's ratio of 2.55), *d*, *l* and *k* are the diameter, outer segment length and absorption coefficient of the photoreceptors, respectively. Due to the difficulty in measuring the pupil aperture on board ship and the great variability in the location of the aphakic gap within lanternfishes [29], we used the lens diameter as a measure for *A* in this study. The absorption coefficient *k* is fixed at 0.035 µm^−1^, which is the average value for vertebrates [49].

The previous equation can now be expressed in a different way;
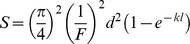
where *F* = F-number = *f*/*A*. Since Matthiessen's ratio states that the focal length in fishes is about 2.55 the radius of the lens [50], [51], then *f* = 1.275*A*, which means that *F* = 1.275 for all the species irrespective of eye size.

The equation can then be written as follows;
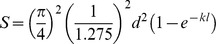



As a result, sensitivity to downwelling light in fishes, following this equation, is completely independent of the size of the lens and is directly proportional to the diameter of the photoreceptors and to a lesser extent to the length of the photoreceptor outer segments. However, it has to be noted that since the sensitivity in this study was only calculated at the level of single photoreceptors, the results will be an underestimate of the true sensitivity of the eye, which is actually set by the level of summation of rods onto ganglion cells, i.e. for a true measure of *S*, *d* in a vertebrate eye should really be the diameter of the ganglion cell's dendritic field (which also ultimately defines the visual pixel size and thus the spatial resolution of that part of the retina). Thus, the values of *S* calculated here may give a false picture of the true situation, and any conclusions based on these values will have to be interpreted carefully.

Sensitivity to bioluminescent flashes (point-like sources, in photons) was estimated using the following formula from Warrant [52] and, Warrant and Locket [53]:
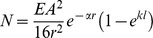



In the equation, *E* is the number of photon emitted at the source, *A* is the pupil diameter, *r* is the distance in meters between the light source and the eye, *α* is the combined attenuation coefficient of bioluminescence (due to scattering and absorption of light by water) and *k* is the photoreceptor absorption coefficient. As for the previous equation, the pupil diameter was replaced by the lens diameter for *A* and *k* was fixed at 0.035 µm^−1^ [49]. *E* was fixed at 10^10^ photons as in Warrant and Locket [53], *r* was set at 1 m and *α* fixed at 0.05 m^−1^ [1]. This equation shows that sensitivity to bioluminescence is directly proportional to the size of the lens and to a lesser extent to the length of the outer segments. However, this equation does not take into account the background space light (i.e. downwelling light), which ultimately limits the maximum distance at which a point source can be detected. In fact, the brighter the background illumination (i.e. higher up in the water column), the harder the point source light will be to visualise, and that independently of the size of the eye – a larger eye (*A* in the equation) will admittedly let in more point source light, but will also let in more background light. Consequently, any *N* sensitivity comparisons between individuals in this study will have to be done cautiously, keeping in mind this caveat. Nevertheless, it is still fair to say that for two fishes at exactly the same depth, *N* will be greater for the fish with the bigger pupil, and in that sense this fish will have greater sensitivity to a point source flash.

### Phylogenetic analyses

While standard statistical analyses assume independence of the samples, this is not true when comparing different species, as more closely related species are expected to be more similar to one another due to sharing a common ancestor. Therefore, all data analyses were performed using phylogenetic comparative analyses to account for the shared history among species [54].

**Figure 2:**
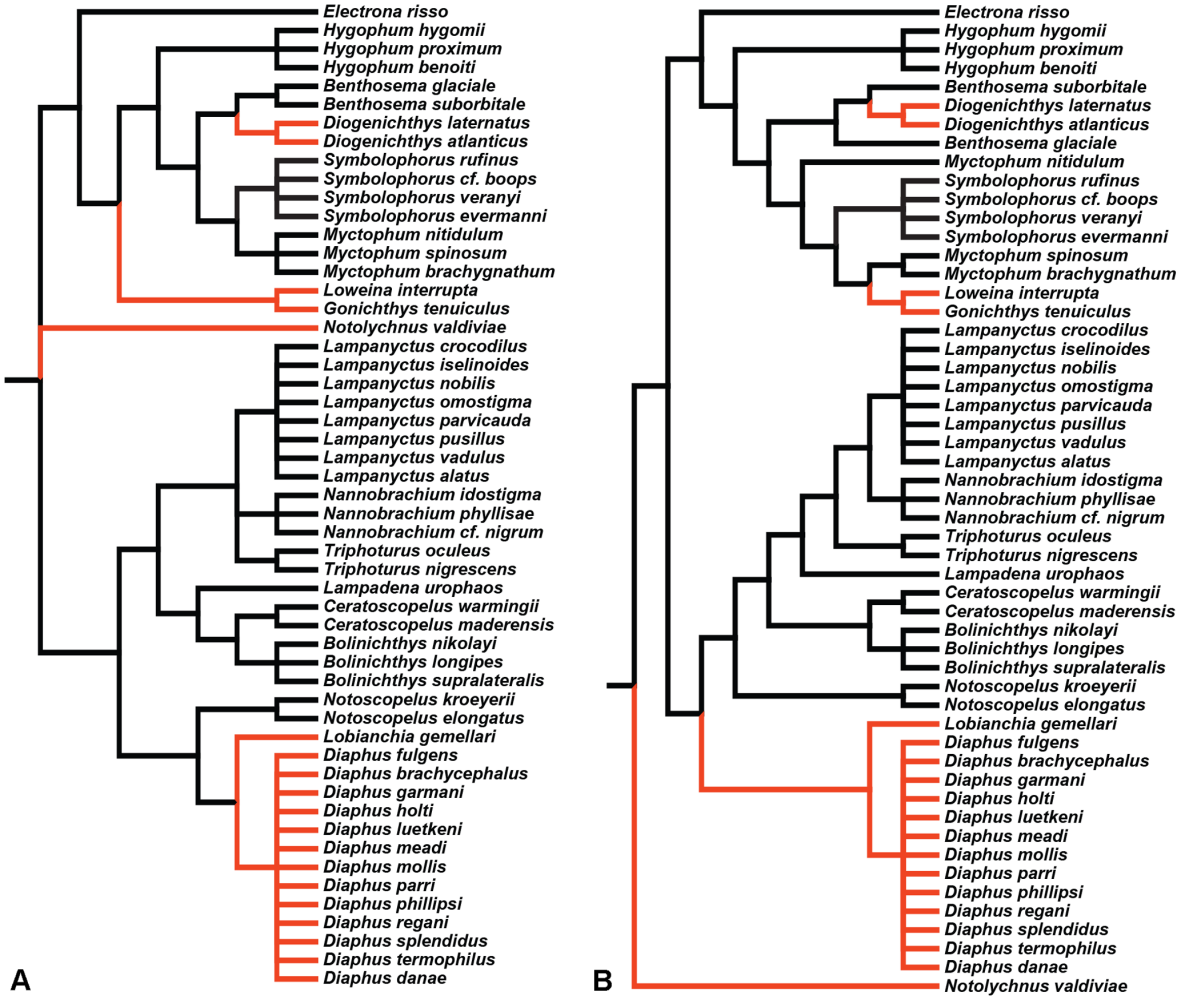
Phylogenetic trees of the Myctophidae family reconstructed from (A) Paxton et al. [56], (B) Poulsen et al. [57]. The red branches indicate the main differences between the two trees. Branch lengths are arbitrarily ultrametricized on the figure. Modified from de Busserolles et al. [27].

The analyses were conducted as described in de Busserolles et al. [27]. Briefly, as no fully resolved phylogeny is currently available for the family Myctophidae, two different phylogenies, A and B (Figure 2), were built using the Mesquite program v. 2.75 [55] based on two different published phylogenies [56], [57].

The main differences between the two phylogenies are the position of the taxon *Notolychnus* and the position of the tribe Diaphini. In fact, in phylogeny B, *Notolychnus* became a sister taxon of all the remaining myctophids, and the Diaphini became a sister tribe of the Lampanyctini. Due to the lack of resolution, both phylogenies are only resolved to the generic level, resulting in several polytomies (i.e. unresolved relationship among species). Unfortunately, the presence of polytomies prevents the application of many phylogenetic analyses. Therefore, to bypass this problem, 100 alternative phylogenies were generated with polytomies randomly resolved to infinitesimally small (10^−6^) branch lengths using the Mesquite program v. 2.75 [55]. Ten of these phylogenies with randomly resolved polytomies were selected at random to perform the different analyses and the result between each of the 10 phylogenies was compared for consistency. Moreover, to fit the statistical requirements for the phylogenetic linear models described below, branch lengths were transformed using Grafen's method [58] with rho transformation set at 2.5 before all analyses. All statistical analyses were performed, using both phylogenies separately, on Log_10_-transformed data with the statistical program R v.2.15.0 (R Foundation for Statistical Computing 2012).

### Ecological data

For the purpose of the statistical analyses, we used the same ecological dataset as in de Busserolles et al. [27], which includes information about the luminous organs and depth distribution patterns of each species examined here. More specifically, the presence-absence of luminous organs (enlarged Dn/Vn, caudal), additional luminous patches, sexual dimorphism in luminous tissues and the type of sexual dimorphism in luminous tissues (Dn/Vn, caudal luminous organs and/or luminous patches) was noted for each species. However, as in de Busserolles et al. [27], the differentiation of the type of sexual dimorphism in our analyses did not show significant differences. As a consequence, only results for the presence/absence of sexually dimorphic features are presented in this study. In terms of depth distribution, each species was assigned a categorised depth range during night and day [27]. The group categories were created in terms of the amount of downwelling light present and resulted in three groups for each time of the day: moderate light level (0–5 m at night, 200–500 m during the day), low light level (5–100 m at night, 500–900 m during the day) and no light (<100 m at night, <900 m during the day).

### Estimating phylogenetic signal

The phylogenetic signal for continuous and discrete traits was estimated with Pagel's lambda (λ) using the package GEIGER in R [59]. Pagel's λ is a measure of the degree of phylogenetic dependence in the data [60], meaning to which degree closely related species are more similar to each other than what is expected by random evolutionary processes. Pagel's λ varies from 0 to 1, with λ value of 1 indicating that traits gradually accumulate changes over time in a Brownian motion process (i.e. random change in any direction) and λ values of 0 indicating that no phylogenetic signal is present and that traits have evolved in response to selective processes. The observed λ value for each trait was compared to λ values of zero and one using likelihood ratio tests with df = 1.

### Phylogenetic linear models

The relationships between each of the morphological traits (eye size, photoreceptor size) and the relationships between the morphological and ecological traits (luminous organs, depth distribution) were all assessed using phylogenetic generalised least squares regressions (PGLS, [61]) with the package APE in R [62]. PGLS regressions estimate a phylogenetic scaling parameter, λ, using maximum likelihood methods to determine the degree of covariance in the residuals of the model, while controlling for phylogenetic effects. This approach also examines whether the scaling parameter λ significantly differs from 0 or 1 using likelihood ratio tests, where λ = 0 indicates no phylogenetic dependence in the data and λ = 1 indicates strong phylogenetic association in the data [60], [61]. PGLS models were first used to assess relationships between eye size and photoreceptor traits (rod diameter, outer and inner segment length). Since eye diameter is strongly correlated with standard length within the family [27], standard length was added as a covariate in all analyses. Finally, phylogenetically controlled multiple regression models were used to assess if photoreceptor length and diameter were related with various ecological parameters when correcting for the effect of eye size and standard length. Since all PGLS results were similar between phylogenies, only the results obtained with phylogeny A are presented throughout.

## Results

### Topographic distribution of photoreceptors

**Figure 3:**
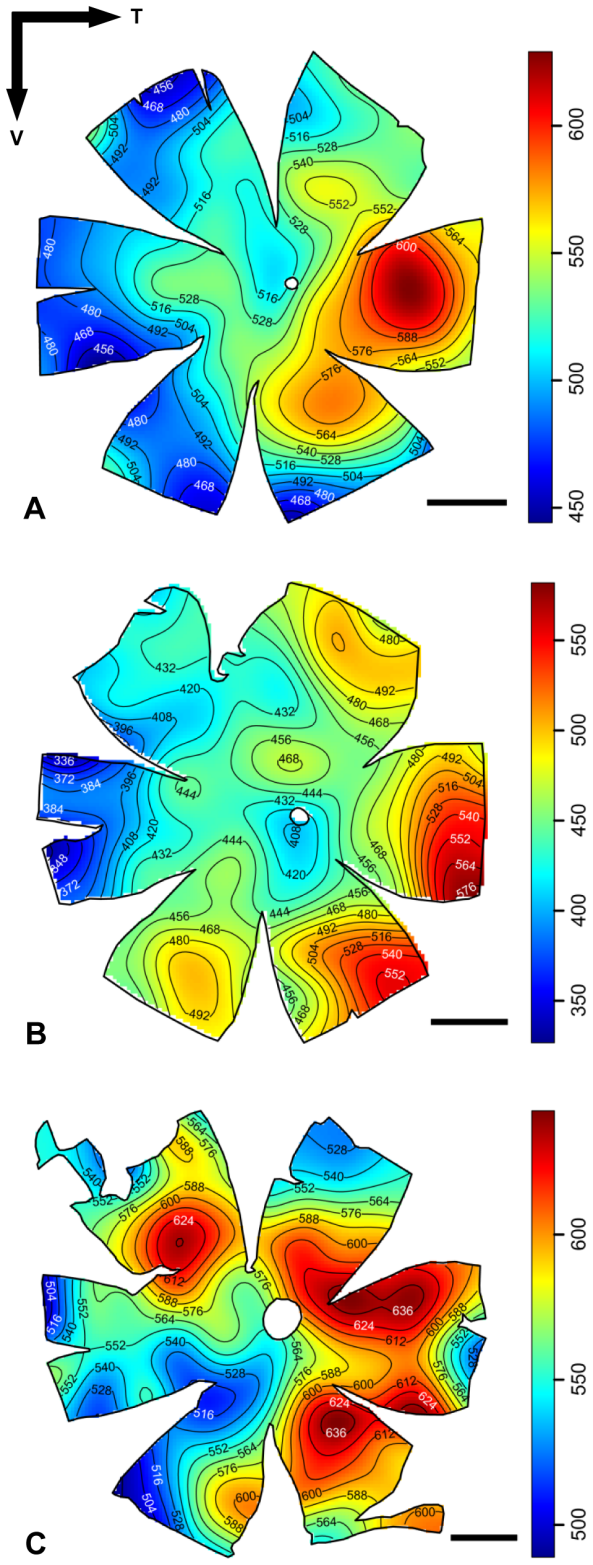
Topographic maps of photoreceptor densities (cells x 10^3^.mm^−2^) for three different individuals of *Bolinichthys longipes*. The black arrows indicate the orientation of the retina. T = temporal, V = ventral. Scale bar = 1 mm. Information about the size of each individual, the stereological parameters used and the quantitative results from the analyses can be found in Table 2 and Table S1.

**Figure 4:**
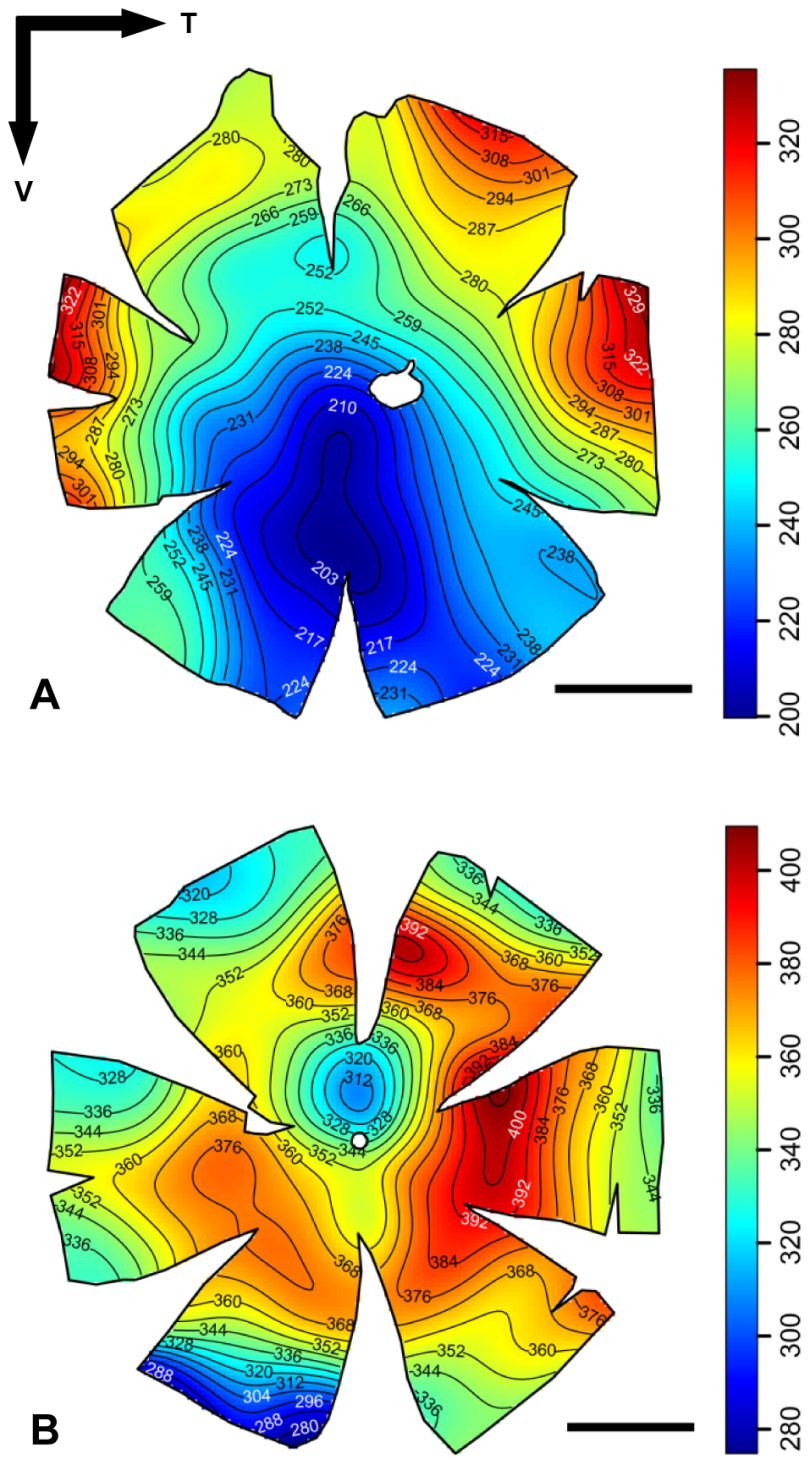
Topographic maps of photoreceptor densities (cells x 10^3^.mm^−2^) for *Diaphus brachycephalus* (A) and *Nannobrachium idostigma* (B). The black arrows indicate the orientation of the retina. T = temporal, V = ventral. Scale bar = 1 mm. Information about the size of each individual, the stereological parameters used and the quantitative results from the analyses can be found in Table 2 and Table S1.

**Figure 5:**
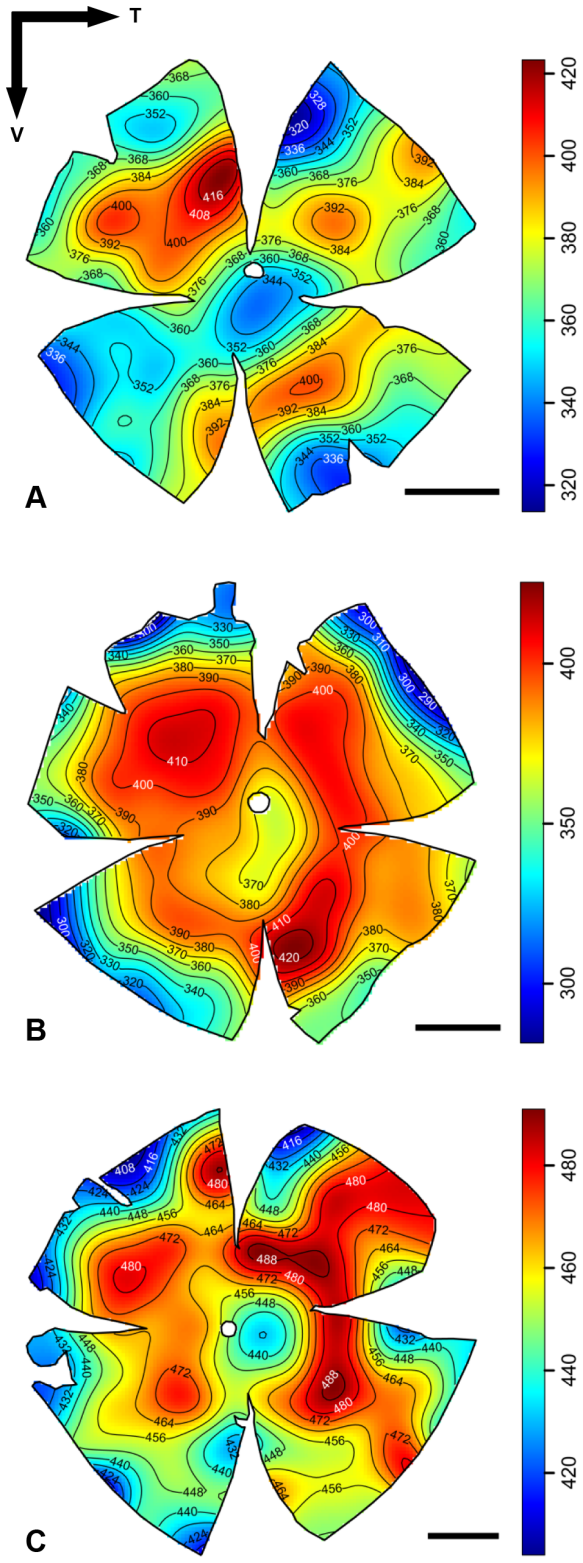
Topographic maps of photoreceptor densities (cells x 10^3^.mm^−2^) for three different individuals of *Lampanyctus parvicauda*. The black arrows indicate the orientation of the retina. T = temporal, V = ventral. Scale bar = 1 mm. Information about the size of each individual, the stereological parameters used and the quantitative results from the analyses can be found in Table 2 and Table S1.

**Figure 6:**
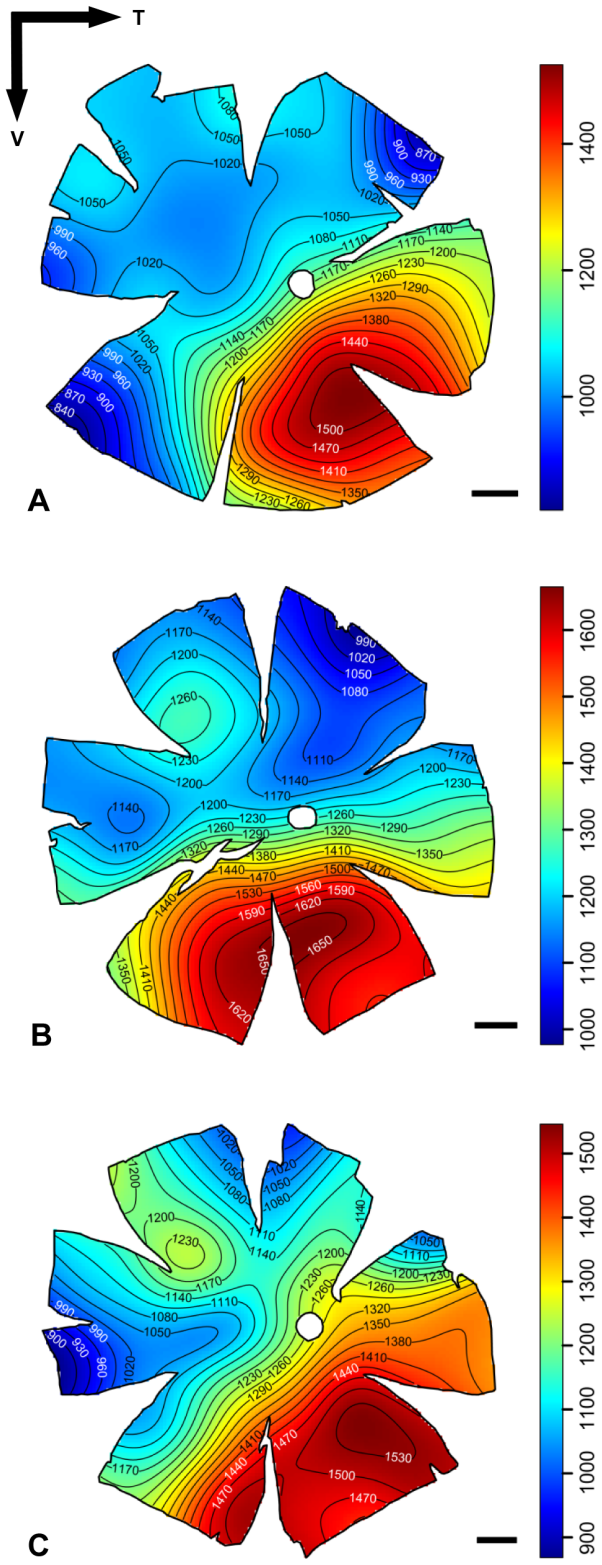
Topographic maps of photoreceptor densities (cells x 10^3^.mm^−2^) for three different individuals of *Myctophum brachygnathum*. The black arrows indicate the orientation of the retina. T = temporal, V = ventral. Scale bar = 1 mm. Information about the size of each individual, the stereological parameters used and the quantitative results from the analyses can be found in Table 2 and Table S1.

Topographic maps of photoreceptor density were constructed for five different species of lanternfishes from wholemounted retinae. Only a single population of rod photoreceptors is present (Figure 1). Rods are densely packed and individually arranged into an hexagonal array (Figure 1), and their density across the retina is heterogeneous, varying greatly between species (Figures 3 to 6). Although some intra-specific variation exists (i.e. *Lampanyctus parvicauda*, Figure 5), consistent patterns can easily be discerned and different specialisations or *areae* of high density can be outlined for each species. Our results reveal the presence of at least three different types of specialisations in lanternfishes: an arch, a ring and a streak-like elongated area. An arch specialisation is observed in two different species, *Bolinichthys longipes* and *Diaphus brachycephalus* (Figures 3 and 4A, respectively). In *B. longipes*, the arch specialisation is present spanning the dorsal-temporal-ventral part of the retina with a peak density of rod photoreceptor cells situated in temporal retina with densities ranging from 650 to 760×10^3^ rods mm^−2^ (Table 2). In *D. brachycephalus*, the arch specialisation is present spanning the nasal-dorsal-temporal part of the retina. A ring specialisation is observed in two species; *Nannobrachium idostigma* and *L. parvicauda* (Figures 4B and 5, respectively) with a peak density of rod photoreceptors ranging from 480 to 570 ×10^3^rods mm^−2^ (Table 2). Finally, a streak-like specialisation is observed in the ventral-temporal part of the retina in one species, *Myctophum brachygnathum* with an elongated decrease of rod photoreceptor density from peak cell densities ranging from 1840 to 2280×10^3^ rods mm^−2^ in the three individuals examined (Figure 6, Table 2).

**Table 2:** Summary of the quantitative data obtained from the optical fractionator method on the wholemount retina.

Species	Individual	Peak cell density (rods x 10^3^.mm^−2^)	Mean cell density(rods x 10^3^.mm^−2^)	Total cell number	Schaeffer CE
*Bolinichthys longipes*	A	750	522	9,185,280	0.030
	B	650	455	9,827,156	0.024
	C	760	575	12,041,724	0.040
*Lampanyctus parvicauda*	A	520	370	4,348,485	0.035
	B	480	374	5,930,100	0.032
	C	570	448	10,218,950	0.026
*Myctophum brachygnathum*	A	1840	1139	61,701,500	0.036
	B	1960 (2280)	1268	83,376,496	0.038
	C	1800 (2120)	1190	85,139,400	0.060
*Diaphus brachycephalus*	A	418	255	3,177,144	0.034
*Nannobrachium idostigma*	B	480	358	3,763,200	0.050

Densities and total cell number are given for each species in addition to the coefficient of error (Schaeffer CE). The peak cell densities in brackets for *M. brachygnathum* were found by sub-sampling.

In terms of topographic changes across the retina, the gradient of rod density is quite shallow for each species. However, *M. brachygnathum* shows a much higher photoreceptor density than the other species with a peak density of 2280×10^3^ rods mm^−2^ compared to peak densities ranging from 418 to 760×10^3^ rods mm^−2^ for the other species (*D. brachycephalus* and *B. longipes*, respectively, Table 2). Intraspecific differences in peak photoreceptor densities are also present and due mainly to differences in the size of the individuals examined, with larger individuals having higher densities across the retina and higher numbers of rods per retina.

**Figure 7:**
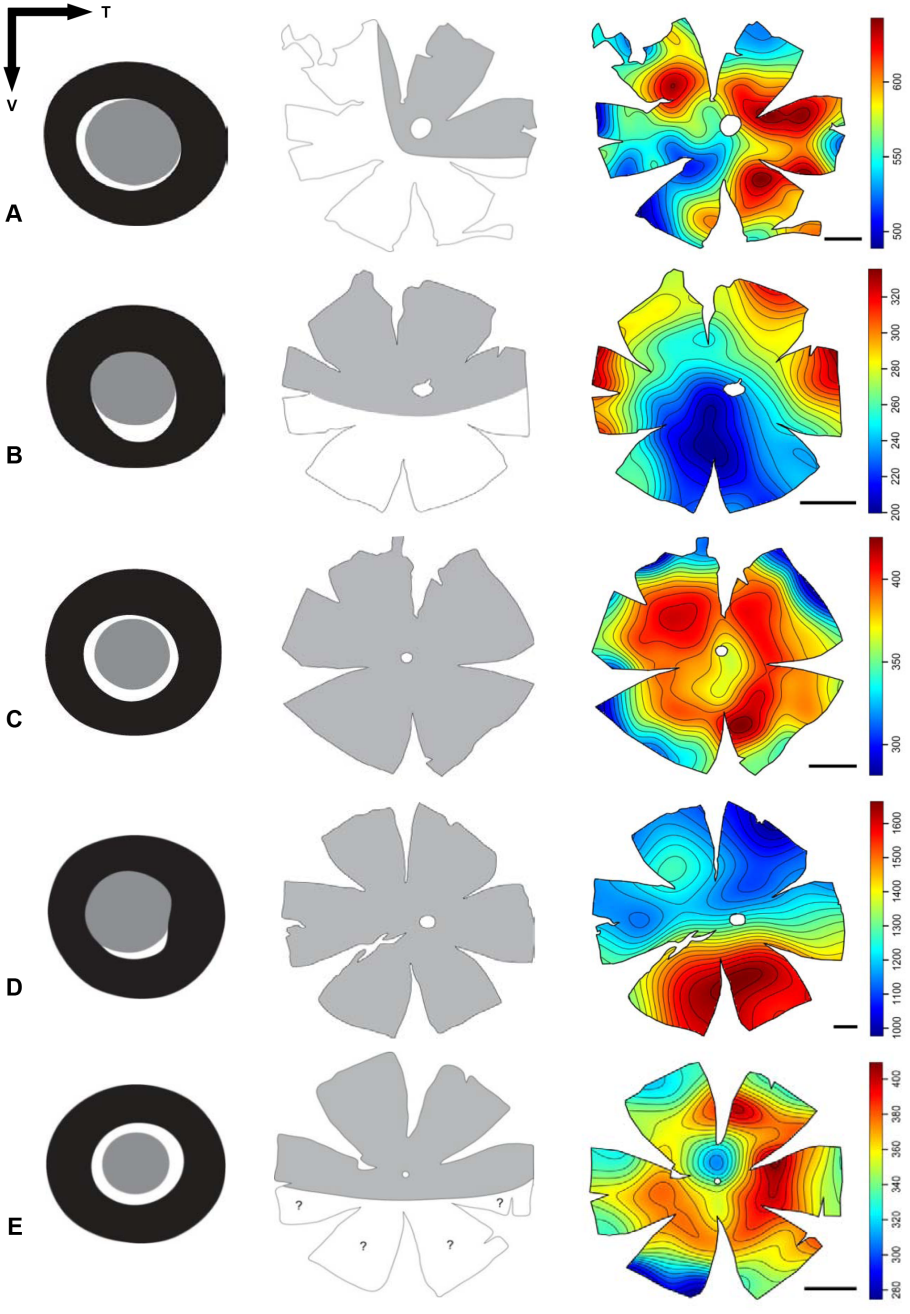
Aphakic gap position (left), tapetum lucidum pattern (middle) and topographic maps of photoreceptor densities (right) for five species of lanternfish. (A) *Bolonichthys longipes*, (B) *Diaphus brachycephalus*, (C) *Lampanyctus parvicauda*, (D) *Myctophum brachygnathum*, (E) *Nannobrachium idostigma*. T = temporal, V = nasal. The aphakic gap is represented in white. The tapetum lucidum, when present, is represented in grey. Scale bar for the maps = 1 mm.

The relationship between the location of the aphakic gap, the presence of a tapetum lucidum and its regional coverage across the retina, and the photoreceptor distribution was visually investigated (Figure 7). Apart from *D. brachycephalus*, which possesses a tapetum lucidum underlying the area of high photoreceptor density, there was no clear relationship between the pattern of tapetal coverage and photoreceptor density. However, the areas of highest photoreceptor density closely align with the position of the aphakic gap, so that the region of the visual field subtended (and therefore sampled) would be increased. However, this was not the case for *M. brachygnathum*, which possesses a peak photoreceptor density directly beneath the aphakic gap, where light striking this retinal region of increased sampling would not be focussed by the lens. Similarly, the two species which possess a ring specialisation of high densities of rod photoreceptors (i.e. *L. parvicauda*, *N. idostigma*, Figures 7 C and D) also have a circumlental aphakic gap, suggesting that large regions of seemingly “specialised” retina are illuminated by unfocussed light, at least on the optical axis. It is therefore clear that the location and function of the aphakic gap may differ between species (Figure 7).

### Morphometric analyses of the photoreceptors

**Figure 8:**
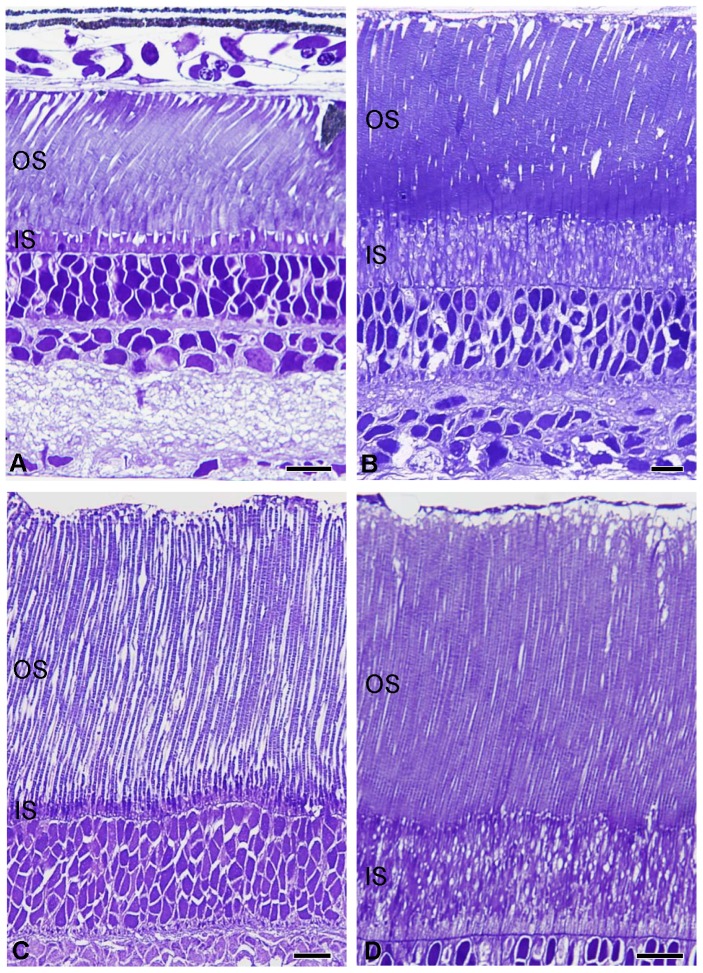
Transverse light microscopy sections through the retina of four species of lanternfish showing the variability in rod length and diameter. (A) *Lampanyctus parvicauda*, (B) *Diaphus brachycephalus*, (C) *Bolinichthys supralateralis*, (D) *Myctophum brachygnathum*. OS = outer segment, IS = inner segment, scale bar = 10 µm.

All the lanternfish species analysed in this study possessed a pure rod retina. A summary of each species' rod morphometric data (length and diameter/width of the inner and outer segment length) and rod density estimates in the central part of the retina, in addition to eye and lens diameter and standard length is given in Table S1. A great variation in rod photoreceptor size is revealed (Figure 8) with species possessing wide rods with small inner segments (i.e. *Lampanyctus parvicauda*, Figure 8A), wide rods with long inner segments (i.e. *Diaphus brachycephalus*, Figure 8B), thin rods with short inner segments (*Bolinichthys supralateralis*, Figure 8C) and thin rods with long inner segments (i.e. *Myctophum brachygnathum*, Figure 8D). In the central part of the retina, rod length varied from 33.3 to 92.9 µm (*Lampanyctus crocodilus* and *Bolinichthys nikolayi*, respectively) with outer segment length ranging from 23.6 µm (*Notoscopelus elongatus*) to 89.0 µm (*Bolinichthys supralateralis*) and inner segment length varying from 3.7 µm (*Lampanyctus crocodilus*) to 22.0 µm (*Gonichthys tenuiculus*). Rod (inner and outer segment) diameter varied from 0.9 to 2.7 µm in *Symbolophorus veranyi* and *Nannobrachium phyllisae*, respectively, and rod density estimates from sections ranged from 194 to 1186×10^3^ mm^−2^ in *Nannobrachium phyllisae* and *Symbolophorus evermanni*, respectively. Density estimates in the central part of the retina from wholemounts and sections give similar results (within 4–10%) with the exception of *Lampanyctus parvicauda* for which estimation from sections underestimated (by∼30%) the rod density compared to the rod density estimated in wholemounts. However, this underestimation could be due to the smaller size of the individual used for sectioning (28.4 mm) compared to the individuals used for wholemounts (54.2 to 65.2 mm).

### Optical sensitivity

**Figure 9:**
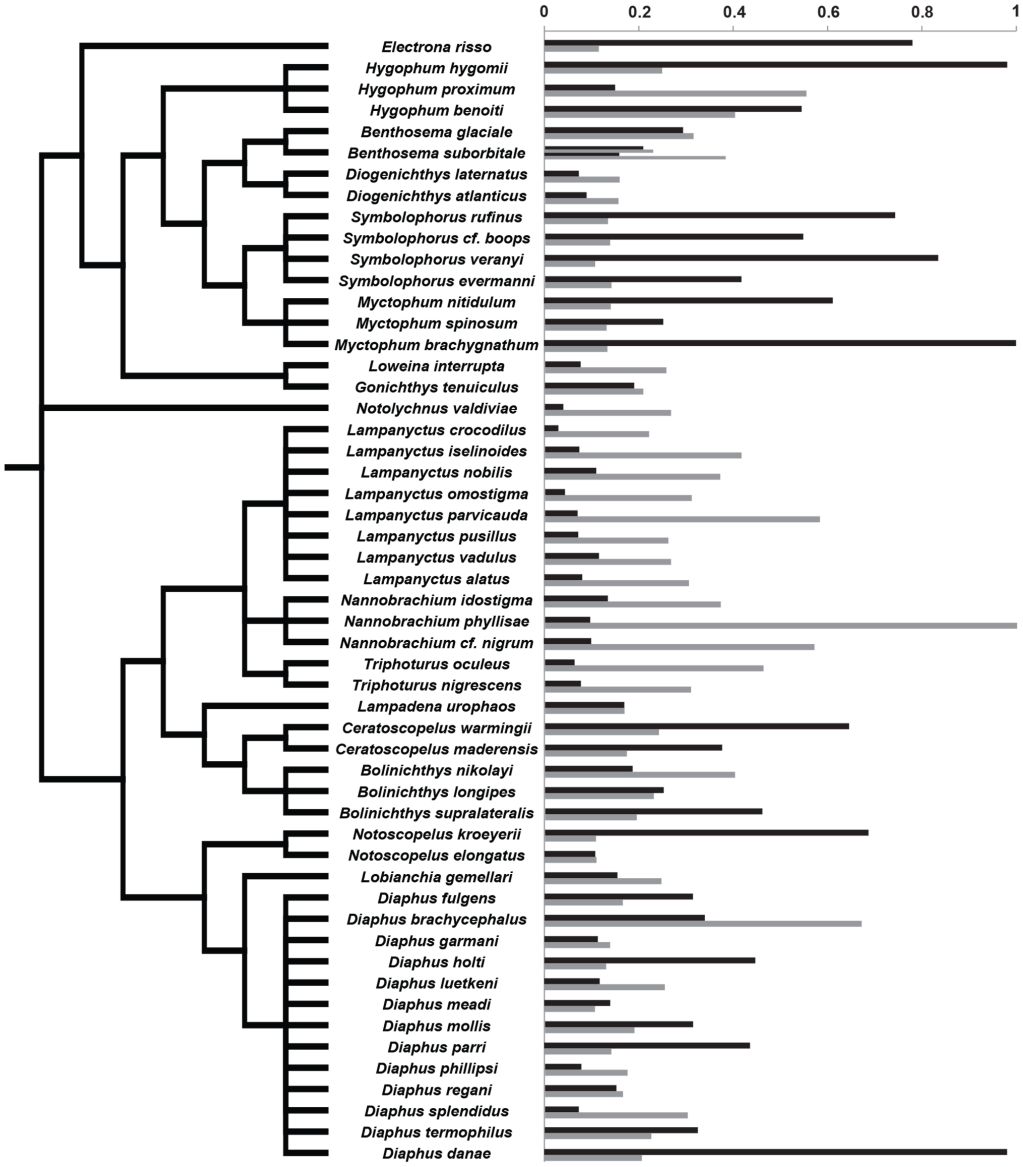
Relative sensitivity to bioluminescence (black bars) and downwelling sunlight (grey bars) for each species of lanternfish analysed in this study. The species are ranked in phylogenetic order following phylogeny A.

Optical sensitivity to downwelling light and bioluminescent flashes was estimated for each species using the lens diameter and the rod measurements taken in the central part of the eye. Sensitivity measures varied greatly between species (Table S1, Figure 9). While some species appear to be particularly sensitive to bioluminescent flashes (*Myctophum sp*, *Symbolophorus sp*, Figure 9), others are more sensitive to downwelling light (*Lampanyctus sp*, *Nannobrachium sp*, Figure 9). A great variation in optical sensitivity was also observed within the same genus, with the genus *Diaphus* representing the most extreme example.

However, these results have to be interpreted carefully. While sensitivity to downwelling light is independent of the size of the eye and principally depends on the diameter of the photoreceptor or, as explained in the Methods, by the diameter of the dendritic fields of the underlying ganglion cells, this is not the case for sensitivity to bioluminescence, which is mainly influenced by the size of the eye. As a result, fishes with larger eyes will automatically have a greater sensitivity to bioluminescent emissions. For this reason, the sensitivity estimations for viewing bioluminescence presented here may not easily be compared without some standardised method of comparing similar-sized individuals and their comparative reliance on each of these two light sources.

### Estimating phylogenetic signal

Estimation of the phylogenetic signal using Pagel's lambda gives relatively similar results with both phylogenies (Table 3). Results show that Dn-Vn and caudal luminous organs have a strong phylogenetic signal and that they gradually accumulated changes over time in a Brownian motion process. On the contrary, no phylogenetic signal was observed for the standard length and the outer segment length variables. An intermediate value of Pagel's lambda was found for the density variable, which, although significantly different from 0 and 1, was closer to 0. All the remaining variables (eye diameter, rod diameter, inner segment length, sensitivity *S* and *N*, luminous patches, sexual dimorphism in luminous tissue and depth distributions) show intermediate values of Pagel's lambda, which, although significantly different from 0 or 1 (except day depth distribution), were generally closer to 1 depending on the phylogenetic tree used.

**Table 3:** Estimates of the phylogenetic signal for each variable using Pagel's Lambda.

Variables	λ (Tree A)	λ (Tree B)
Eye diameter	0.88^<0.001, <0.001^	0.83^0.002, <0.001^
Standard length	<0.001^1, <0.001^	<0.001^1, <0.001^
Residuals eye/SL	0.95^<0.001, <0.001^	0.93^<0.001, <0.001^
Rod diameter	0.90^0.003, <0.001^	0.92^0.01, <0.001^
IS length	0.78^<0.001, <0.001^	0.92^<0.001, <0.001^
OS length	0.03^0.72, <0.001^	<0.001^1, <0.001^
Sensitivity S	0.92^0.001, <0.001^	0.95^0.003, <0.001^
Sensitivity N	0.88^<0.001, <0.001^	0.85^0.002, <0.001^
Rod density	0.11^<0.001, <0.001^	0.09^<0.001, <0.001^
Dn/Vn organs	1^<0.001, 1^	1^<0.001, 1^
Caudal luminous organs	1^<0.001, 1^	1^<0.001, 1^
Luminous patches	0.97^<0.001, <0.001^	0.98^<0.001, <0.001^
Luminous tissue sexual dimorphism	0.92^<0.001, <0.001^	0.96^<0.001, <0.001^
Day depth	0.95^0.53, <0.001^	0.79^0.38, <0.001^
Night depth	0.79^<0.001, <0.001^	0.74^<0.001, <0.001^

The results are presented for one of the ten randomly selected polytomy resolved trees for the two different phylogenies. A λ value of 1 indicates that the trait gradually accumulates changes over time in a Brownian motion process. A λ values of 0 indicates that no phylogenetic signal is present and that traits have evolved in response to selective processes. The superscript values are likelihood ratio tests different from 0 and 1. Sample size is 53 for all variables except day depth (50).

### Relationship among morphometric traits

A phylogenetic linear regression shows that rod diameter is negatively correlated with eye diameter (PGLS, n = 53, R^2^ = 0.18, *t-value* = -2.842, *P* = 0.006), indicating that species with small eyes have larger rods and vice-versa. No other relationships between eye diameter and the other photoreceptor traits (inner and outer segment length) could be identified as being statistically significant using PGLS.

### Relationship between morphometric and ecological traits

The phylogenetically controlled multiple linear regression models revealed relationships between the photoreceptor traits and some of the ecological variables analysed in this study (Table 4). Our results show that rod diameter is negatively correlated with the presence of luminous patches (Table 4) and positively correlated with night depth distribution (Table 4). These results indicate that species that do not possess any additional luminous patches and that venture to deeper depths at night have larger rod photoreceptors and vice-versa. Similar results are found for the sensitivity *S* (to downwelling light) with species without luminous patches (Table 4) and species with a deeper distribution profile at night (Table 4) having a greater sensitivity to downwelling light.

**Table 4:** Regression models of several visual traits with different predictor variables when controlling for phylogeny (PGLS).

Trait	*λ*	Predictor variables	*β*	*t*	*P*
Rod diameter	<0.001^ns, *^	Eye diameter	−0.073	−0.677	0.502
		Standard length	−0.070	−0.562	0.577
		Dn/Vn Luminous organs	−0.055	−1.131	0.264
		Caudal luminous organs	−0.058	−1.155	0.255
		**Luminous patches**	**−0.071**	**−2.209**	**0.033**
		Luminous tissue sexual dimorphism	−0.040	−1.139	0.261
		Day depth	−0.011	−0.539	0.592
		**Night depth**	**0.071**	**2.242**	**0.030**
Sensitivity S	<0.001^ns, *^	Eye diameter	−0.179	−0.809	0.423
		Standard length	−0.136	−0.540	0.592
		Dn/Vn Luminous organs	−0.193	−1.149	0.058
		Caudal luminous organs	−0.171	−1.680	0.101
		**Luminous patches******	**−0.135**	**−2.066**	**0.045**
		Luminous tissue sexual dimorphism	−0.062	−0.860	0.395
		Day depth	−0.039	−0.917	0.365
		**Night depth**	**0.170**	**2.624**	**0.012**
Inner segment length	<0.001^ns, *^	Eye diameter	0.120	0.629	0.533
		Standard length	0.219	1.008	0.319
		Dn/Vn Luminous organs	0.066	0.775	0.443
		Caudal luminous organs	−0.140	−1.592	0.119
		Luminous patches	−0.075	−1.327	0.192
		Luminous tissue sexual dimorphism	0.068	1.092	0.281
		Day depth	0.044	1.193	0.240
		**Night depth**	**−0.117**	**−2.102**	**0.042**
Outer segment length	<0.001^ns, *^	Eye diameter	−0.070	−0.532	0.598
		Standard length	−0.035	−0.231	0.818
		**Dn/Vn Luminous organs**	**−0.192**	**−3.278**	**0.002**
		Caudal luminous organs	−0.106	−1.798	0.084
		Luminous patches	0.045	1.173	0.247
		Luminous tissue sexual dimorphism	0.049	1.145	0.259
		Day depth	−0.047	−1.855	0.071
		**Night depth**	**0.078**	**2.024**	**0.049**
Rod density	<0.001^ns, *^	Eye diameter	−75.59	−0.294	0.770
		Standard length	−407.40	−1.392	0.171
		Dn/Vn Luminous organs	70.76	0.616	0.541
		Caudal luminous organs	63.84	0.541	0.592
		Luminous patches	46.06	0.608	0.547
		**Luminous tissue sexual dimorphism**	**228.03**	**2.730**	**0.009**
		Day depth	−50.38	−1.009	0.319
		Night depth	−128.87	−1.713	0.094
Eye diameter	0.909^*, *^	Standard length	0.932	10.708	<0.001
		Dn/Vn Luminous organs	−0.002	−0.027	0.979
		Caudal luminous organs	−0.140	−1.810	0.077
		Luminous patches	0.001	0.034	0.973
		Luminous tissue sexual dimorphism	0.077	1.749	0.088
		Day depth	0.003	0.154	0.878
		Night depth	0.043	1.133	0.264
Sensitivity N	0.933^*,*^	Standard length	1.766	9.379	<0.001
		Dn/Vn Luminous organs	−0.146	−1.123	0.268
		Caudal luminous organs	−0.319	−1.800	0.079
		Luminous patches	−0.067	−0.834	0.409
		Luminous tissue sexual dimorphism	0.140	1.500	0.141
		Day depth	0.028	0.606	0.548
		Night depth	0.099	1.215	0.231

The results are identical and independent of the phylogeny used. Standard length was added as a covariate in the models. λ  =  phylogenetic scaling parameter, the superscript * after the parameter λ indicates whether the parameter was significantly different from 0 (first position) and from 1 (second position) in the likelihood tests, β = partial regression slope. In bold are the significant results. The sampling size was 50.

Phylogenetically controlled multiple linear regression models also revealed relationships between photoreceptor length and ecological traits. The results indicate that the inner segment length is negatively correlated with the night depth distribution (Table 4) meaning that species living deeper at night have smaller inner segments and vice-versa. Finally, outer segment length was negatively correlated with the presence/absence of head luminous organs (Table 4) and positively correlated with night depth distribution (Table 4) indicating that species having Dn/Vn luminous organs and a shallow distribution at night have smaller outer segments and vice-versa.

A positive relationship was also found between rod photoreceptor density and the presence of sexual dimorphism (Table 4) where species with sexually dimorphic luminous tissues have greater rod densities. Finally, no statistically-significant relationships were found between sensitivity *N* (to bioluminescence) and any of the ecological variables using PGLS (Table 4).

## Discussion

The aim of this study was to assess the variability in photoreceptor characteristics within a large range of species of lanternfishes with different ecological traits to assess the influence(s) of both ecology and phylogeny on the evolution of their visual system. This study follows on from a previous investigation looking at eye size variation in lanternfishes in relation to their ecology [27]. The aim of the de Busserolles et al. [27] study was borne from the assumption that there is a gradual change in the visual scene in the mesopelagic zone with depth and that this will ultimately result in a great diversity in eye size [1], [52], [63]. They hypothesised that lanternfishes with a deeper distribution range and/or that have less reliance on bioluminescence (i.e. having less luminous tissues) will have smaller eye sizes. This relationship was found not to exist for lanternfishes, where relative eye size could not be linked to any of the ecological variables tested but was instead strongly influenced by phylogeny [27]. The present investigation was initiated to explore what other visual characteristics may account for the high levels of variability in behaviour in an environment, which would appear to be heavily reliant on visual signals for survival.

### Topographic variations in sampling the ambient light environment

Topographic analyses of photoreceptor and ganglion cell distributions are very useful in providing information about the visual ecology of a species by identifying areas of the visual field of high importance (i.e. area of high cell densities, [64]–[67]). Despite photoreceptors playing a major role in the process of vision by collecting light information and initiating phototransduction, topographic analyses of photoreceptor densities are quite sparse in teleosts compared to ganglion cell analyses and are non-existent in deep-sea teleosts. To our knowledge, this is the first analysis of photoreceptor distribution across the retina in any deep-sea species of bony fishes.

Since the acuity is limited by the amount of light available, areas of high photoreceptor density usually match the peak(s) in ganglion cell density [68], [69]. Topographic analyses of ganglion cell distribution (not including amacrine cells) have been examined in three species of lanternfishes from the genus *Lampanyctus*, and reveal a poorly specialised retina, showing a nearly uniform distribution of cells within the ganglion cell layer [70], [71]. Conversely, results from this study, on different species from a range of genera, show a great diversity in visual specialisations with different species having distinct areas of high photoreceptor density (with respect to both peak density and the shape of the specialised acute zone) i.e., an arch in *Bolinichthys longipes* and *Diaphus brachycephalus*, a ring in *Lampanyctus parvicauda* and *Nannobrachium idostigma* and a streak-like elongated area in *Myctophum brachygnathum*. Although a topographic analysis of ganglion cells was not performed for these species, it is likely that their distribution would match that of the photoreceptors [68], [69], thereby indicating possible interspecific differences in visual capability. Moreover, the fact that the position of the aphakic gap matches the photoreceptor distribution in most species examined emphasises the importance of a specific area of the visual field in any visually-guided behaviours in each species.

The retinal morphology of teleost fishes is highly diverse and correlates very well with habitat complexity and behavioural ecology [65], [66], [72]. In the mesopelagic zone, different ecological tasks are more likely to influence each species' photoreceptor topography. Two types of light stimuli can be detected in the mesopelagic zone, downwelling sunlight and bioluminescence. Assessing the intensity of downwelling light is essential to a species' ability to maintain a particular depth during the day, camouflage its silhouette by counterillumination, trigger vertical migration, set circadian rhythms and/or detect the presence of prey or predator from below. In contrast, assessing the intensity and frequency of bioluminescent signals will be crucial for detecting other individuals (prey, predator, mate) at deeper depths, where bioluminescent cues predominate (i.e. in the North Atlantic, 90% of the individuals below 500 m produce bioluminescence, [6]). As most lanternfish vertically migrate [73]) and possess photophores used for counterillumination [25], interspecific differences in photoreceptor topography will most likely be due to differences in how each species interact with prey, predators and/or mates. Several lanternfish species possess sexually dimorphic luminous tissues that are thought to play a role in sexual communication [74]. However, topographic analyses of the retinae of both males and females will have to be conducted to reveal any sexual dimorphism with respect to the location of these retinal “acute” zones and how they are used in visually-guided behaviour(s) underlying reproduction.

*Diaphus brachycephalus* possesses a dorsal arch, a dorsal tapetum and a ventral aphakic gap. All these specialisations may work together to enhance the capture of photons of light emanating from below, most likely to detect bioluminescence signals in the lower part of the visual field. A similar scenario may apply to *Bolinichthys longipes,* which possesses a temporal arch extending dorsally and ventrally, a dorso-temporal tapetum and a ventro-nasal aphakic gap. In this species, the eye is specialised to light capture in the frontal and ventral visual fields. In *Myctophum brachygnathum*, the area of peak photoreceptor density is situated in the ventral part of the retina, providing higher sampling of light signals emanating from above. If the distribution of both the photoreceptor and ganglion cell populations are in register, this ventral-temporal acute zone will enhance the detection of a silhouette against the lighter background of the upper mesopelagic zone. Although not optimised for receiving a focussed image, the ventral aphakic gap in this species might facilitate the detection of bioluminescent signals (prey, predator or mate) situated below the fish, within the increased visual field produced by this ventral extension of the pupillary aperture.

*Lampanyctus parvicauda* and *Nannobrachium idostigma* do not possess any isolated specialisations enhancing visual capabilities within a particular part of the visual field. Instead, both species possess a ring specialisation in addition to a circumlental aphakic gap, which would enhance the chance of photon capture in all directions. The lack of a specialisation mediating acute vision within a specific part of the visual field may indicate that these two species do not rely on vision as much as other species and rely more on other sensory systems, as appears to be the case for few other myctophid species [75]. It is also possible that those species are visual generalists, interacting with other individuals to avoid predation but feeding opportunistically and targeting a wide range of prey items [76], [77]. Myctophids are mainly zooplankton consumers (i.e. copepods, euphausids, amphipods) with a high diversity of organisms comprising their diet [76]–[82]. Lanternfishes also provide food for a wide range of higher level organisms like teleost fishes [83], [84], cephalopods i.e. squid [85], [86], seabirds [87], [88], and mammals [77], [89], [90]. Interspecific differences in the diet and methods of predation could explain differences in the topographic distribution of photoreceptor cells. Unfortunately, these data are not yet available for the species analysed in this study and would have to be considered in any future interpretations.

### Different strategies for optimising light capture by retinal photoreceptors

Our results highlight a great diversity in photoreceptor design within the Myctophidae at all levels. Differences are evident in terms of photoreceptor distribution (as discussed above), photoreceptor dimensions (length and diameter) and density.

Overall, rod outer segments in lanternfishes are not particularly long compared to other deep-sea species with similar retinal organisation (i.e. a single bank of photoreceptors, [71]), with a maximum length of 89 µm recorded for *Bolinichthys longipes* (this study) compared to others species which possess rod outer segments over 100 µm in length i.e. 150 µm in *Platytroctes apus* [91] and 170 µm in *Sternoptix* sp. [92]. Some species of myctophids possess relatively small outer segments i.e. *Diaphus phillipsi*, comparable to the rod photoreceptors in goldfishes [93].

Rod outer segment diameter in myctophids is also relatively small compared to what has been recorded for other deep-sea fishes [9], [94]. In some myctophid species, rods are extremely small i.e. <1 µm, *Symbolophorus rufinus*, making them one of the narrowest photoreceptors found in both vertebrates and invertebrates, including insects [95] and approaching the optical limits for photon capture. These minute rod diameters also equate to very high photoreceptor densities, reaching peaks of 1186×10^3^ mm^−2^ in *Symbolophorus evermanni* and 2280×10^3^ mm^−2^ in *Myctophum brachygnathum*. These high rod densities far exceed what has previously been recorded for any other deep-sea fishes, including those species that possess a deep convexiclivate fovea [71], and also greatly exceed the highest recorded peak rod density for any vertebrate, i.e 1000×10^3^ mm^−2^ in the oilbird, *Steatornis caripensis* [96]. Even at the lowest end of the rod densities recorded for the myctophid species examined in this study, i.e. 194×10^3^ mm^−2^, *Nannobrachium phillisae*, the values are still higher than those found in bottom dwelling deep-sea fish [71], sharks [97] and shallow water teleosts [98]. Furthermore, around half of the species analysed in this study showed peak rod densities higher than those recorded for nocturnal birds and mammals with a similar retinal organisation, i.e. a single bank of photoreceptors with peak density of 341×10^3^ rods mm^−2^ in the great horned owl, *Bubo virginianus* [99] and 500×10^3^ rods mm^−2^ in the cat, *Felis domesticus* [100].

Very high photoreceptor densities usually denote a high level of summation between photoreceptors, interneurons and ganglion cells resulting in high sensitivity [53]. Peak densities of ganglion cells were not investigated in this study, although if one considers the highest recorded density of ganglion cells found for a lanternfish (7.4×10^3^ rods mm^−2^ in *Lampanyctus ater*, [71] and the highest photoreceptor density reported in this study (2286×10^3^ mm^−2^, *Myctophum brachygnathum*), the summation/convergence ratio could potentially be as high as 309 photoreceptors to one ganglion cell. In broad terms, high levels of summation are indicated by the presence of a relatively thick outer nuclear layer (high numbers of rod nuclei) and a thin inner nuclear/ganglion cell layer (low number of bipolar cells and ganglion cells), which seems to be the case for a large number of lanternfish species [29].

In terms of optical sensitivity, each species seems to be specialised for the detection of a specific signal (downwelling light or bioluminescence), which might reflect different behaviours (Figure 9). While the determination of sensitivity estimates to downwelling light is straightforward, the sensitivity estimations to bioluminescence are biased due to the influence of eye size (as outlined in the Methods and Results sections). In fact, fishes with larger eyes will automatically have a greater sensitivity to bioluminescent emissions. For this reason, the sensitivity estimations for viewing bioluminescence presented here may not easily be compared without some standardised method of comparing similar-sized individuals (i.e. relative eye size). Moreover, comparisons of optical sensitivities between species have to be made cautiously since the influence of several specialisations were not accounted for in the calculations i.e. the presence of a tapetum lucidum and an aphakic gap in some species. The presence of a tapetum lucidum and an aphakic gap would augment photon capture by indirectly increasing both the outer segment length (given the reflection of light rays incident on the tapetal plates) and the size of the pupillary aperture, respectively. One or both of these ocular specialisations occur in a number of species [29] and will undoubtedly increase sensitivity to both downwelling light and bioluminescent light flashes, in a specific part of the visual field. For example, some species like *Lampanyctus* sp and *Nannobrachium* sp possess very small eyes and large photoreceptors. The large size of these photoreceptors means that they are particularly sensitive to downwelling light, but the size of the eye is limiting in terms of sensitivity to bioluminescence. However, both species possess specialisations, such as a circumlental aphakic gap and a tapetum lucidum to overcome these issues.

The high level of interspecific variability in strategies for optimising light capture by the photoreceptors within the Myctophidae make the task of assessing visual capabilities and sensitivity quite challenging, especially if one wants to use a standardised method.

### The influence(s) of the photoreceptors on ecological variation in visual behaviour

Results from the phylogenetic comparative analyses highlighted several relationships between photoreceptor characteristics and the ecological variables tested (depth distribution and luminous tissue patterns). The results of these models are discussed in detail below.

#### Rod diameter and sensitivity

Our results reveal that species with no luminous patches and a deeper depth distribution at night possess larger rods. Although a negative relationship between rod diameter and relative eye size was found in a previous model with species with smaller eyes having larger rods (this study), this relationship disappears when all the ecological traits are added to the model. This shows that even though rod diameter is influenced by the relative eye size of a species and that both characters are strongly influenced by phylogeny (Table 4), it is the species' ecology that drives this component of the visual system in lanternfishes.

Since rod diameter and, to a lesser extent, outer segment length both determine sensitivity to downwelling light in lanternfishes in this study, it is not surprising to find similar relationships between sensitivity to downwelling light and ecological traits to the ones found with rod diameter. Results indicate that species without any luminous patches and with a deeper distribution profile at night possess higher sensitivity to downwelling light. Even though downwelling light at night (starlight, moonlight), is considerably dimmer in intensity than sunlight (10^−6^ - 10^−7^ dimmer, [1], [101]), starlight illumination, in coastal waters, may still be enough to allow vision below 200 m [102]. Our results indicate that species living deeper at night, where less downwelling light is present, have adapted to this environment by possessing a greater sensitivity to such a light.

As discussed previously, rod size in lanternfishes is a good predictor of rod density with larger rods denoting lower rod densities. Although ganglion cell distribution and density was not investigated in this study, a relatively thin inner nuclear layer compared to the outer nuclear layer suggests a high summation ratio in most species [29]. As a result, rod densities may be a good proxy of summation in lanternfishes, although this will have to be verified in further studies. If this is the case, in addition to possessing larger rods and therefore, a greater sensitivity to downwelling light, deeper living species might also possess lower summation ratios. While high summation greatly improves photon catch by providing visual channels that view large solid angles of space, it occurs at the expense of spatial and temporal resolution. In deeper water at night, where less downwelling light is present, bioluminescent light flashes will appear a lot brighter due to a reduced background space light, thereby creating a point-like image on the retina. In that situation, large visual channels are not necessary and lower levels of summation will be sufficient to view the signal and keep image resolution optimised [52], [103]. Consequently, higher sensitivity to downwelling light (i.e. larger rods) and lower levels of spatial summation (i.e. lower rod density) might allow the eyes of deeper living species to be better adapted to visualise their environment at night. We therefore consider that the relationship between rod size and each species' depth distribution at night to be the most significant in terms of lanternfish visual ecology. However, this hypothesis will have to be verified in future studies with the analysis of ganglion cell density and distribution for the same species examined in this study. Moreover, further analyses are needed to understand the role of the luminous patches in the visual behaviour of myctophids. Although there is a possible role in intraspecific communication given the presence of sexual dimorphism, the function(s) of the luminous patches in lanternfishes remains unclear.

#### Outer segment length and density

Mesopelagic fishes possess several specialisations to enhance sensitivity compared to their shallow water counterparts. Within the Myctophidae, these specialisations include an increase in outer segment length and high densities of rod photoreceptors. A large variability in both of these parameters also indicates different levels of sensitivity. As per the formulae of Land [48] and Warrant and Nilsson [49], an increase in outer segment length will augment both sensitivity to downwelling light and bioluminescence. PGLS results indicate that species with no Dn/Vn luminous organs and with a deeper depth distribution at night have longer outer segments. Several hypotheses have been proposed for the function of the Dn/Vn organs in myctophids. They may be used 1. As a head torch, creating an extended scene of light in their frontal visual field to search for prey [7], [52], [104], 2. To compare the intensity of their own photophore emissions with the levels of downwelling sunlight in order to camouflage the silhouette of their body when viewed from below [28] and/or 3. For intraspecific communication in sexually dimorphic species [74]. At deeper depths at night, sensitivity to downwelling light is not that useful and therefore increases in outer segment length is more likely an adaptation to better visualise bioluminescent signals.

PGLS results also reveal that species with a sexual dimorphism in luminous tissues possess higher rod densities. As previously discussed, high photoreceptor density in lanternfishes, may denote high levels of spatial summation [29], an adaptation that would increase sensitivity. The fact that species with sexually dimorphic luminous tissues might possess more sensitive eyes may support a long standing hypothesis proposed by a range of authors [26], [74], [105], that bioluminescence is used in intraspecific communication in lanternfishes.

#### Inner segment length

Our results show that species with a deeper depth distribution at night possess shorter rod inner segments. Photoreceptor inner segments contain mitochondria, the metabolic drivers of the cell. In addition to their clear metabolic function, mitochondria may also have an optical function by guiding the light toward the outer segments [106]. A shorter inner segment could therefore indicate lower energetic requirements (by the presence of less mitochondria) or less reliance on light guiding. In both cases, the reason as to why depth would influence rod inner segment length is currently unknown. However, since mitochondria could be packed in different ways depending on the width of the inner segment, variability in mitochondrial density between lanternfish species could be investigated in future analyses to help in understanding interspecific variation. There may also be differential effects of pressure on metabolic function that could be investigated.

## Conclusions

A great diversity in the visual system of the Myctophidae is observed at the level of the photoreceptors, the first stage of retinal processing. This study provides the first analysis of photoreceptor distribution in any deep-sea teleost and reveals clear interspecific differences in visual specialisations (areas of high rod photoreceptor density), indicating potential interspecific differences in interactions with prey, predators and/or mates. A great diversity in photoreceptor design (length and diameter) and density is also present. Overall, the myctophid eye is very sensitive compared to other teleosts and each species seem to be specialised for the detection of a specific signal (downwelling light or bioluminescence), potentially reflecting different visual demands for survival. Differences in photoreceptor characteristics could be related to differences in ecological variables (i.e. depth distribution at night), highlighting the importance of ecological factors on the evolution of the visual system in lanternfishes.

## Supporting Information

Table S1 Summary of eye and retinal measurements for 53 species of lanternfishes. Sensitivities to downwelling light (S) and bioluminescence (N) and rod photoreceptor density estimations are also given. IS = inner segment, OS = outer segment, ø  =  diameter.
(DOC)
