# Supplementary material for: The Influence of Photoreceptor Size and Distribution on Optical Sensitivity in the Eyes of Lanternfishes (Myctophidae)
Source: PLoS One. 2014 Jun 13;9(6):e99957. doi: 10.1371/journal.pone.0099957 (PMC4057366; doi:10.1371/journal.pone.0099957)
Supplement: Table S1 — Summary of eye and retinal measurements for 53 species of lanternfishes. Sensitivities to downwelling light (S) and bioluminescence (N) and rod photoreceptor density estimations are also given. IS = inner segment, OS = outer segment, ø = diameter. (DOC) [file pone.0099957.s001.doc]

**Table S1. Summary of eye and retinal measurements for 53 species of lanternfishes.** Sensitivities to downwelling light (S) and bioluminescence (N) and rod photoreceptor density estimations are also given. IS=inner segment, OS=outer segment, ø = diameter.

| **Species** | **Cruise** | **SL (mm)** | **Eye ø (mm)** | **Lens ø (mm)** | **OS length (μm)** | **IS length (μm)** | **Rod ø (μm)** | **Density estimate**  **(x10^3^.mm^-2^)** | **Sensitivity S (μm^2^.sr )** | **Sensitivity N (photons)** |
| --- | --- | --- | --- | --- | --- | --- | --- | --- | --- | --- |
| *Benthosema glaciale* | 8 | 42.0 | 4.6 | 1.8 | 40.3 | 9.6 | 1.6 | 433 ± 82 | 0.75 | 1377 |
| *B. suborbitale* long rods | 9 | 28.2 | 3.0 | 1.3 | 75.1 | 7.8 | 1.3 | 643 ± 122 | 0.55 | 990 |
| short rods |  |  |  |  | 34.3 | 3.7 | 1.9 | 346 ± 66 | 0.92 | 746 |
| *Bolinichthys longipes* | 4 | 35.0 | 3.8 | 1.5 | 58.7 | 9.2 | 1.3 | 430 ± 82 | 0.55 | 1182 |
| *B. nikolayi* | 3 | 27.5 | 2.9 | 1.2 | 87.4 | 5.5 | 1.6 | 313 ± 59 | 0.96 | 871 |
| *B. supralateralis* | 2 | 41.2 | 4.2 | 2.0 | 89.0 | 7.3 | 1.1 | 666 ± 127 | 0.46 | 2160 |
| *Ceratoscopelus maderensis* | 8 | 52.0 | 5.1 | 2.1 | 34.3 | 6.5 | 1.3 | 520 ± 99 | 0.41 | 1763 |
| *C. warmingii* | 1 | 60.8 | 5.9 | 2.5 | 49.2 | 12.0 | 1.4 | 457 ± 87 | 0.58 | 3026 |
| *Diaphus brachycephalus* | 3 | 35.2 | 4.3 | 1.8 | 50.3 | 16.7 | 2.3 | 233 ± 44 | 1.60 | 1595 |
| *D. danae* | 3 | 92.2 | 8.2 | 3.3 | 34.7 | 16.6 | 1.4 | 379 ± 72 | 0.49 | 4609 |
| *D. fulgens* | 2 | 41.2 | 3.4 | 1.8 | 42.7 | 12.0 | 1.2 | 659 ± 125 | 0.40 | 1478 |
| *D. gamani* | 4 | 33.1 | 2.5 | 1.0 | 50.2 | 15.6 | 1.0 | 732 ± 139 | 0.33 | 532 |
| *D. holti* | 8 | 40.0 | 5.1 | 2.1 | 45.0 | 13.4 | 1.0 | 840 ± 160 | 0.31 | 2099 |
| *D. luetkeni* | 3 | 38.3 | 2.4 | 1.1 | 53.6 | 6.9 | 1.5 | 437 ± 83 | 0.61 | 552 |
| *D. meadi* | 9 | 28.3 | 3.3 | 1.2 | 36.2 | 10.2 | 1.0 | 873 ± 166 | 0.26 | 657 |
| *D. mollis* | 3 | 39.5 | 4.0 | 1.8 | 46.0 | 12.9 | 1.2 | 767 ± 146 | 0.45 | 1474 |
| *D. parri* | 2 | 51.0 | 5.6 | 2.1 | 45.5 | 15.0 | 1.1 | 530 ± 683 | 0.34 | 2050 |
| *D. phillipsi* | 3 | 27.4 | 2.2 | 1.0 | 27.7 | 14.0 | 1.3 | 425 ± 55 | 0.42 | 362 |
| *D. regani* | 4 | 40.8 | 2.5 | 1.2 | 55.9 | 11.5 | 1.1 | 731 ± 79 | 0.39 | 711 |
| *D. splendidus* | 3 | 34.1 | 2.1 | 0.8 | 60.5 | 8.7 | 1.5 | 532 ± 55 | 0.72 | 335 |
| *D. termophilus* | 3 | 48.3 | 4.2 | 1.8 | 40.5 | 13.2 | 1.4 | 540 ± 65 | 0.54 | 1525 |
| *Diogenichthys atlanticus* ♀ | 9 | 20.1 | 2.1 | 0.8 | 43.3 | 6.9 | 1.1 | 879 ± 184 | 0.35 | 312 |
| *Diogenichthys atlanticus* ♂ | 9 | 21.4 | 2.7 | 1.0 | 31.5 | 6.9 | 1.2 | 843 ± 146 | 0.37 | 413 |
| *D. laternatus* ♀ | 6 | 31.1 | 3.2 | 1.3 | 42.8 | 8.9 | 1.2 | 478 ± 62 | 0.43 | 780 |
| *D. laternatus* ♂ | 6 | 22.1 | 2.2 | 0.9 | 38.1 | 9.7 | 1.2 | 552 ± 54 | 0.38 | 347 |
| *Electrona risso* | 8 | 46.0 | 7.0 | 3.0 | 31.8 | 5.4 | 1.0 | 805 ± 111 | 0.28 | 3666 |
| *Gonichthys tenuiculus* | 6 | 41.3 | 2.9 | 1.4 | 40.0 | 22.0 | 1.3 | 973 ± 228 | 0.50 | 890 |
| *Hygophum benoiti* | 8 | 45.0 | 6.2 | 2.3 | 45.1 | 11.3 | 1.8 | 271 ± 43 | 0.96 | 2561 |
| *H. hygomii* | 9 | 57.3 | 7.5 | 3.1 | 44.7 | 9.4 | 1.4 | 362 ± 54 | 0.60 | 4607 |
| *H. proximum* | 6 | 26.2 | 3.2 | 1.2 | 44.7 | 10.3 | 2.1 | 195 ± 29 | 1.32 | 700 |
| *L. urophaos* | 3 | 40.0 | 2.8 | 1.3 | 42.5 | 13.1 | 1.2 | 498 ± 64 | 0.40 | 790 |
| *Lampanyctus alatus* | 9 | 43.2 | 2.1 | 0.9 | 51.5 | 5.2 | 1.5 | 452 ± 58 | 0.73 | 376 |
| *L. crocodilus* | 8 | 31.0 | 1.7 | 0.6 | 29.6 | 3.7 | 1.5 | 294 ± 47 | 0.53 | 138 |
| *L. iselinoides* | 6 | 34.3 | 2.0 | 0.8 | 59.4 | 5.2 | 1.7 | 264 ± 27 | 0.99 | 341 |
| *L. nobilis* | 2 | 37.5 | 2.1 | 1.0 | 46.3 | 4.1 | 1.7 | 405 ± 56 | 0.89 | 516 |
| *L. omostigma* | 6 | 27.8 | 1.9 | 0.6 | 48.0 | 7.4 | 1.6 | 362 ± 50 | 0.74 | 198 |
| *L. parvicauda* | 6 | 28.4 | 1.8 | 0.9 | 36.4 | 5.6 | 2.3 | 280 ± 29 | 1.40 | 324 |
| *L. pusillus* | 8 | 37.0 | 2.1 | 0.82 | 53.1 | 14.2 | 1.4 | 259 ± 54 | 0.63 | 337 |
| *L. vadulus* | 3 | 37.4 | 2.2 | 1.02 | 58.5 | 8.3 | 1.4 | 225 ± 39 | 0.64 | 539 |
| *L. gemellari* | 3 | 32.7 | 2.1 | 1.24 | 45.9 | 11.3 | 1.4 | 595 ± 64 | 0.59 | 728 |
| *Loweina interrupta* | 9 | 25.0 | 2.1 | 0.88 | 41.8 | 16.3 | 1.5 | 381 ± 46 | 0.61 | 354 |
| *Myctophum brachygnathum* | 2 | 67.7 | 7.6 | 3.15 | 45.5 | 14.7 | 1.0 | 1078 ± 226 | 0.32 | 4699 |
| *M. nitidulum* | 6 | 85.4 | 7.1 | 2.47 | 44.9 | 20.7 | 1.1 | 1050 ± 168 | 0.34 | 2874 |
| *M. spinosum* | 4 | 39.6 | 3.9 | 1.63 | 39.1 | 15.1 | 1.1 | 866 ± 105 | 0.31 | 1177 |
| *Nannobrachium cf. nigrum* | 3 | 52.3 | 2.5 | 0.97 | 52.7 | 4.4 | 2.1 | 246 ± 34 | 1.37 | 471 |
| *N. idostigma* | 6 | 72.2 | 3.9 | 1.16 | 45.6 | 5.4 | 1.7 | 312 ± 40 | 0.89 | 638 |
| *N. phyllisae* | 6 | 50.4 | 2.2 | 0.95 | 52.1 | 5.7 | 2.7 | 194 ± 27 | 2.39 | 450 |
| *Notolychnus valdiviae* | 3 | 19.5 | 1.5 | 0.60 | 55.9 | 13.4 | 1.4 | 484 ± 92 | 0.64 | 184 |
| *Notoscopelus elongatus* | 8 | 47.0 | 3.2 | 1.23 | 23.6 | 14.3 | 1.1 | 548 ± 104 | 0.26 | 505 |
| *N. kroeyerii* | 7 | 101.2 | 6.1 | 2.74 | 36.5 | 14.8 | 1.0 | 852 ± 148 | 0.26 | 3221 |
| *Symbolophorus cf. boops* | 6 | 72.0 | 6.0 | 2.12 | 37.6 | 14.2 | 1.1 | 782 ± 116 | 0.34 | 1954 |
| *S. evermanni* | 2 | 59.0 | 5.1 | 2.81 | 39.0 | 12.7 | 1.1 | 1186 ± 206 | 0.32 | 3496 |
| *S. rufinus* | 2 | 69.0 | 6.5 | 2.30 | 48.5 | 17.4 | 1.0 | 871 ± 231 | 0.33 | 2569 |
| *S. veranyi* | 8 | 85.0 | 6.7 | 2.94 | 41.1 | 13.7 | 0.9 | 751 ± 120 | 0.26 | 3917 |
| *Triphoturus nigrescens* | 3 | 35.4 | 2.0 | 0.85 | 50.9 | 10.7 | 1.5 | 303 ± 39 | 0.74 | 357 |
| *T. oculeus* | 6 | 33.9 | 2.1 | 0.80 | 42.5 | 5.6 | 1.9 | 318 ± 59 | 1.11 | 295 |
